# The *ParaHox* gene *Gsx* patterns the apical organ and central nervous system but not the foregut in scaphopod and cephalopod mollusks

**DOI:** 10.1186/s13227-015-0037-z

**Published:** 2015-12-29

**Authors:** Tim Wollesen, Sonia Victoria Rodríguez Monje, Carmel McDougall, Bernard M. Degnan, Andreas Wanninger

**Affiliations:** Department of Integrative Zoology, Faculty of Life Sciences, University of Vienna, 1090 Vienna, Austria; School of Biological Sciences, The University of Queensland, Brisbane, QLD 4072 Australia

**Keywords:** Brain, Cephalopoda, Evolution, Development, *Hox*, Homeobox genes, Invertebrate, Lophotrochozoa, Mollusca, Ontogeny, Scaphopoda, Lophotrochozoa

## Abstract

**Background:**

It has been hypothesized that the *ParaHox* gene *Gsx* patterned the foregut of the last common bilaterian ancestor. This notion was corroborated by *Gsx* expression in three out of four lophotrochozoan species, several ecdysozoans, and some deuterostomes. Remarkably, *Gsx* is also expressed in the bilaterian anterior-most central nervous system (CNS) and the gastropod and annelid apical organ. To infer whether these findings are consistent with other mollusks or even lophotrochozoans, we investigated *Gsx* expression in developmental stages of representatives of two other molluscan classes, the scaphopod *Antalis entalis* and the cephalopod *Idiosepius notoides*.

**Results:**

*Gsx* is not expressed in the developing digestive tract of *Antalis entalis* and *Idiosepius notoides*. Instead, it is expressed in cells of the apical organ in the scaphopod trochophore and in two cells adjacent to this organ. Late-stage trochophores express *Aen*-*Gsx* in cells of the developing cerebral and pedal ganglia and in cells close to the pavilion, mantle, and foot. In postmetamorphic specimens, *Aen*-*Gsx* is expressed in the cerebral and pedal ganglia, the foot, and the nascent captacula. In early squid embryos, *Ino*-*Gsx* is expressed in the cerebral, palliovisceral, and optic ganglia. In late-stage embryos, *Ino*-*Gsx* is additionally expressed close to the eyes and in the supraesophageal and posterior subesophageal masses and optic lobes. Developmental stages close to hatching express *Ino*-*Gsx* only close to the eyes.

**Conclusions:**

Our results suggest that *Gsx* expression in the foregut might not be a plesiomorphic trait of the Lophotrochozoa as insinuated previously. Since neither ecdysozoans nor deuterostomes express *Gsx* in their gut, a role in gut formation in the last common bilaterian ancestor appears unlikely. *Gsx* is consistently expressed in the bilaterian anterior-most CNS and the apical organ of lophotrochozoan larvae, suggesting a recruitment of *Gsx* into the formation of this organ in the Lophotrochozoa. The cephalopod posterior subesophageal mass and optic ganglia and the scaphopod pedal ganglia also express *Gsx*. In summary, *Gsx* expression only appears to be conserved in the anterior-most brain region during evolution. Accordingly, *Gsx* appears to have been recruited into the formation of other expression domains, e.g., the apical organ or the foregut, in some lophotrochozoans.

## Background

The *Hox* and *ParaHox* gene clusters are considered to be derived from a hypothetical *ProtoHox* cluster by duplication [1]. Both belong to the homeobox gene family and exhibit highly conserved amino acid sequences in phylogenetically distantly related animals [1, 2]. In the majority of bilaterians investigated, it has been shown that *Hox* genes are expressed in tempo-spatial collinearity during development, in particular in neuroectodermal domains [2, 3]. Cephalopod and gastropod mollusks were among the first examples among bilaterians that apparently do not exhibit such a collinear mode of *Hox* gene expression [4, 5]. Tempo-spatial collinear expression of the three *ParaHox* genes has also been proposed for the last common bilaterian ancestor [1]. It has been hypothesized that *Gsx* was expressed in the foregut, *Xlox* in the midgut, and *Cdx* in the hindgut in the last common bilaterian ancestor [1, 6]. While *Xlox* expression in the midgut and *Cdx* expression in the hindgut was found in various bilaterians, no *Gsx* expression has been reported in the foregut of any deuterostome representative to date [1, 6]. This was explained by the fact that the blastopore does not develop into the prospective mouth in deuterostomes. Deuterostomes instead evolved a new mouth and hence *Gsx* might have lost its role in patterning the anterior-most region of the digestive tract. Interestingly, the deuterostome hemichordate *Ptychodera flava*, for example, does express *Gsx* around the blastopore, however, apparently not in the digestive tract of subsequent developmental stages [7]. Holland anticipated that protostome invertebrates may show *Gsx* expression in the foregut since their blastopore usually does become the future mouth [1, 6].

Data on the ecdysozoan and lophotrochozoan condition show, however, an ambiguous picture. While all ecdysozoans investigated so far do not appear to express *Gsx* in their digestive tract, the situation in lophotrochozoans is less clear (Table 1). The annelids *Platynereis dumerilii* and *Nereis virens* and the gastropod *Gibbula varia* express *Gsx* in their foregut [12–14], while the annelid *Capitella teleta* does not [15]. Comparisons with the condition in the Cnidaria, the putative bilaterian sister group, do not appear to contribute to inferring the ancestral state of *Gsx* expression in the Bilateria since the different germ layers cannot be homologized convincingly among the Cnidaria and the Bilateria. In addition, *Gsx* expression patterns are not consistent among cnidarians. In the planula larvae of *Nematostella vectensis*, *Clytia hemisphaerica*, and *Podocoryne carnea* [9–11], *Gsx* is expressed in the endoderm, while it is expressed in the ectoderm of the planula of *Acropora millepora* [8].

**Table 1:** *Gsx* gene expression domains in metazoan developmental stages as revealed by in situ hybridization experiments

Super-phylum/clade/*species*	Name of *Gsx ortholog*	*Gsx* expression domains	References
Cnidaria			
*Acropora millepora*	*Cnox*-*2Am*	Planula larva Ectodermal cells along the oral/aboral body axis (rare in oral region)	[8]
*Nematostella vectensis*	*Anthox2*	Planula larva Posterior endoderm, i.e., prospective oral end Developing mesenteries (ectoderm), Late planula larva Columnar ectodermal cells in tentacle buds Oral ectoderm	[9]
*Clytia hemisphaerica*	*Gsx Ch*	Planula and embryos Endodermal cells in oral and aboral region	[10]
*Podocoryne carnea*	*Gsx*	Planula Anterior and posterior endoderm	[11]
Lophotrochozoa			
Gastropoda *Gibbula varia*	*Gva*-*Gsx*	Trochophore Bilateral pair of 4-5 cells in dorso-median episphere (anlagen of cerebral ganglia?) Pair of each three sensory cells in apical organ Cells around stomodeum Pre-torsional veliger Two apical tuft cells and sensory cup cells of apical organ Cells around mouth opening Ventral portion of nascent digestive gland Post-torsional competent veliger Ventral portion of digestive gland Cells around mouth opening Cells at ventral border of the yolk-filled cells Cells in cerebral ganglia anlagen Cells in foregut close to radula anlage Postlarval development Posterior radula sac	[12]
Scaphopoda *Antalis entalis*	*Aen*-*Gsx*	Early-stage trochophore 2 cells each in the lateral episphere on both sides 1 cell each lateral to the anus on both sides Mid-stage trochophore 1 pair of cells in the apical organ and another pair lateral to latter 1 cell each lateral to the anus on both sides 1 cell each in posterolateral mantle on both sides Late-stage trochophore Several cells in the region of the cerebral and pedal ganglia and ventral foot Metamorphic competent trochophore Several cells in the region of the cerebral and pedal ganglia, the ventral foot, and the captacula Postmetamorphic individual Several cells in the region of the cerebral and pedal ganglia, the ventral foot, and the captacula	Present study
Cephalopoda *Idiosepius notoides*	*Ino*-*Gsx*	Stage 19–20 Cerebral, optic, and palliovisceral ganglia Stage 23 Cerebral, optic, and palliovisceral ganglia Stage 25 Inferior frontal lobes, precommissural lobes, anterior and posterior basal lobes, inferior buccal lobes, Stage 26 Inferior frontal lobes, precommissural lobes, anterior and posterior basal lobes, inferior buccal lobes, peduncle lobes, and optic lobes Stages 27–30 Region around eyes	Present study
Annelida			
*Platynereis dumerilii*	*Pdu*-*Gsx*	Trochophore Few cells in apical hemisphere in apical organ and cerebral ganglia Cells of ventral plate during differentiation of trunk CNS Two bilateral clusters of cells close to stomodeum Setiger larva Cells in midgut and posterior foregut	[13]
*Nereis virens*	*Nvi*-*Gsh*	Trochophore Bilateral cell clusters in dorso-median episphere Multiple bilaterally expression domains in dorsolateral episphere that persist during later larval development Stomodeum Early juveniles Large cells at dorsal part of head at position of adult eyes No expression in older juveniles	[14]
*Capitella teleta*	*CapI*-*Gsx*	Embryo (stages 5-8) Small domain of anterior CNS	[15]
Ecdysozoa			
Arthropoda			
*Drosophila melanogaster*	*ind* (*intermediate neuroblasts defective*)	Intermediate column cells of developing CNS In intermediate ectodermal domain of antennal segment Dorsal ectodermal region of the ocular region	[16, 17]
*Tribolium castaneum*	*Tc*-*ind*	Intermediate column cells of developing CNS	[18]
Deuterostomia			
Echinodermata *Strongylocentrotus purpuratus*	*Sp*-*Gsx*	Gastrula and subsequent larval stage Two bilateral neuroectodermal domains	[19]
*Patiria miniata*	*Pm*-*Gsx*	Provided as maternal message with no zygotic activation in subsequent developmental stages	[20]
Hemichordata *Ptychodera flava*	*PfGsx*	Gastrula Cells around blastopore of gastrula (disappear in tornaria larva)	[7]
Chordata			
*Branchiostoma floridae*	*AmphiGsx*	Anterior CNS	[21, 22]
*Ciona intestinalis*	*Ci*-*gsx*	Anterior CNS	[23]
*Mus musculus*	*Gsh*-*1, Gsh*-*2*	*Gsh*-*1* CNS (neural tube, hindbrain, mesencephalon, diencephalon) *Gsh*-*2* CNS (forebrain, midbrain, hindbrain)	[24–26]
*Danio rerio*	*Gsh*-*1*	*Gsh*-*1* Early embryo In hindbrain rhombomeres late embryo In mesencephalon, diencephalon, and intermediate spinal cord	[27]
*Oryzias latipes*	*Ol*-*Gsh 1*	*Gsh 1* Neuroectoderm (spinal cord, dorsal rhombencephalon, optic tectum, dorsal diencephalon, hypothalamus, rostral telencephalon)	[28]
*Xenopus tropicalis*	*Gsh*-*1*, *Gsh*-*2*	*Gsh*-*1* and *Gsh*-*2* Anterior neural plate/CNS	[29]

Few studies have been carried out on *Gsx* expression in juvenile or adult bilaterians

*Gsx* is also involved in the development of the CNS in bilaterians, and it is expressed in distinct cells of the apical organ in the gastropod mollusk *G. varia* and the annelid *P. dumerilii* (Table 1; [12, 13]). In addition, *Gsx* expression was also found in the radula sac, a molluscan evolutionary novelty [12]. Recent phylogenomic analyses on mollusks have revived a classical hypothesis placing the Aculifera, i.e., the worm-shaped and spicule-bearing aplacophorans and the eight-shelled polyplacophorans, as a sister group to the Conchifera [30–32]. The Conchifera is an anatomically diverse clade comprising scaphopods, gastropods, bivalves, monoplacophorans, and cephalopods. Until now, conchiferan interrelationships are unsettled, and attempts to infer the evolution of their body plans are scarce (c.f. [31, 32]; but see [33, 34]).

**Fig. 1:**
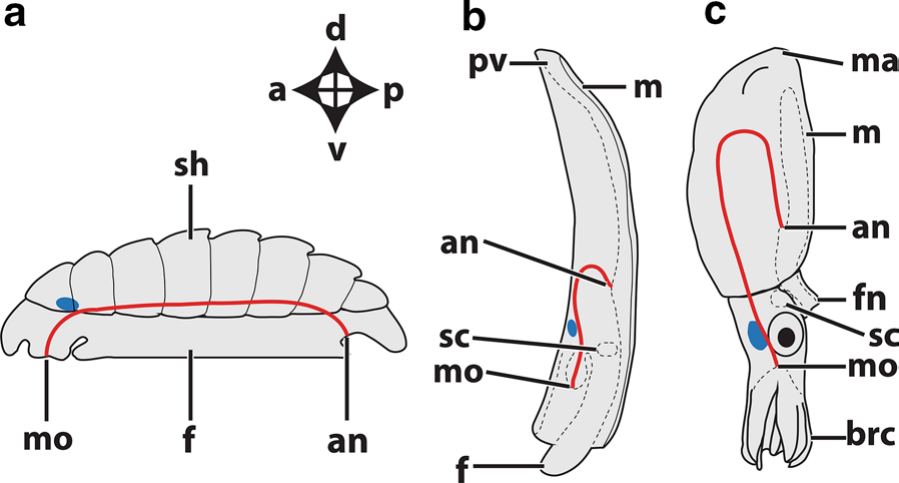
Dorso-ventral and antero-posterior body axes in adult mollusks. Dorsal (*d*)–ventral (*v*), anterior (*a*)-posterior (*p*) axes indicate the orientation. The (anlagen of the) cerebral ganglia/commissure (*blue*) are located anteriorly, while the statocysts (*sc*) are located posteriorly (statocysts are absent in polyplacophorans). The trajectory of the digestive tract is* lined* in* red*. **a** An adult polyplacophoran with a pronounced antero-posterior axis, a dorsal shell (*sh*) and ventral mouth (*mo*) and foot (*f*). **b** The mouth and foot of the adult scaphopod *Antalis entalis* are located ventrally, while the scaphopod pavilion (*pv*) lies dorsally. **c** The mouth and brachial crown (*brc*) of the adult cephalopod *Idiosepius notoides* are located ventrally, while the mantle apex (*ma*) is located dorsally. *an* anus, *fn* funnel, *m* mantle and *pt* prototroch

The present study deals with two conchiferans, the scaphopod *Antalis entalis* Jeffreys 1869 and the cephalopod squid *Idiosepius notoides* Berry, 1921 (Fig. 1). Adult scaphopods and cephalopods exhibit a pronounced dorso-ventral body axis as opposed to the majority of bilaterians that exhibit a pronounced antero-posterior body axis (Fig. 1). In adult scaphopods, the mouth and foot are located ventrally, while the pavilion (i.e., the mantle cavity opening on the opposite side) marks the dorsal pole (Fig. 1b). In adult cephalopods, the funnel and (parts of) the circumoral brachial crown are considered to be homologous to the foot of other mollusks [35] (Fig. 1c). The brachial crown and the funnel define the ventral side, while the mantle apex is located dorsally (Fig. 1c). Thus, the dorso-ventral axis constitutes the major body axis in these animals. In both clades, the cerebral ganglia are located anteriorly (labeled blue in Fig. 1), while the statocysts are located at the posterior pole (dashed circles in Fig. 1b, c).

### Ontogeny of the scaphopod *Antalis entalis* and the cephalopod *Idiosepius notoides*

**Fig. 2:**
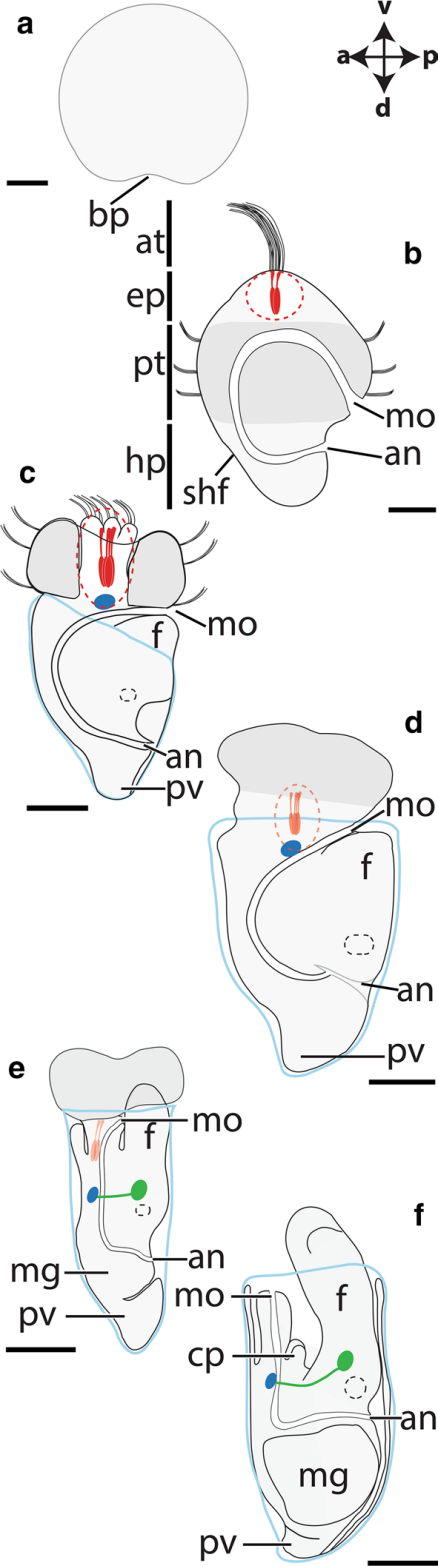
Ontogeny of the scaphopod *Antalis entalis*. All lateral views and dorsal (*d*)–ventral (*v*), anterior (*a*)–posterior (*p*) axes indicate the orientation. Note the prototroch (*dark gray*), the statocysts (*black dashed encircled*), the apical organ (*red dashed circle*) with serotonin-like immunoreactive cells (*red*) and apical tuft (*at*), the cerebral ganglia (*blue*) and the pedal ganglia with connectives (*green*). The mouth (*mo*) is located ventrally to the foot (*f*), while the anus (*an*) is located ventrally to the pavilion (*pv*; dorsal mantle opening). The *light blue line* outlines the shell. **a** Gastrula with blastopore (bp) (12 hpf). **b** The early-stage trochophore (14 hpf) exhibits an episphere (*ep*) with an apical organ with two serotonin-like immunoreactive cells. The prototroch (*pt*) divides the episphere from the hyposphere (*hp*). In the anterior hyposphere, the shell field (*shf*) develops. **c** Mid-stage trochophore (21 hpf) possesses four serotonin-like immunoreactive cells in addition to two lateral cells (not shown) that do not belong to the apical organ. **d** Late-stage trochophore (63 hpf). **e** Metamorphic competent trochophore (70 hpf). **f** Postmetamorphic and settled specimen (114 hpf). Data on serotonin-like immunoreactive cells and on the location of the CNS derive from [36–38, 40]. *cp* captacula, *mg* midgut gland. *Scale bars* 50 µm

In the scaphopod, *A. entalis* gastrulation occurs at 12 h after fertilization (hpf) at 21–23 °C (Fig. 2a). At 14 hpf, a trochophore larva develops that exhibits an episphere with an apical organ and tuft (red dashed circles in Fig. 2). The episphere is divided from the hyposphere by a prototroch (Fig. 2b; see also [36–38]). The gastropod trochophore resembles the latter, but while the apical region develops into the prospective anterior region in gastropods, it develops into the prospective ventral region in scaphopods (see scaphopod condition in Fig. 2; [12, 36–38]). The blastopore of the gastrula develops into the mouth in *A. entalis* and lecithotrophic early-stage trochophore larvae already possess a through-gut with mouth and anus (Fig. 2a, b). The apical organ exhibits two serotonin-like immunoreactive cell somata (labeled red in Fig. 2b), and the nascent shell field is located in the anterior region of the hyposphere (Fig. 2b; [39]). The apical organ of mid-stage trochophore larvae (21 hpf) possesses four serotonin-like immunoreactive cells that are located next to two lateral cells that do not belong to this sensory organ (Fig. 2c). The episphere including the apical organ migrates in direction of the dorsal side and the cerebral ganglia develop below the latter and ventrally to the esophagus (Fig. 2c) [40]. In mid-stage trochophore larvae, the statocysts become visible in the foot (black dashed circles in Fig. 2c), and the dorsal-most region of the mantle, the pavilion, serves as second opening of the mantle cavity. In late-stage trochophore larvae (63 hpf) and advanced developmental stages, the dorso-ventral body axis elongates considerably and the foot grows out into ventral direction (Fig. 2d). The apical organ migrates in dorsal direction and most probably disappears with all serotonin-like immunoreactive cells in metamorphic competent trochophore larvae (Fig. 2e). The cerebral ganglia are located anteriorly (blue domain in Fig. 2e) and connect to the pedal ganglia that are located ventrally to the statocysts (green domain in Fig. 2e). During metamorphosis, trochophores settle and are able to retract their prototroch and foot into the shell. Postmetamorphic individuals do not exhibit a prototroch and possess two captacula anlagen. These are the forerunners of the multiple cephalic tentacles that are used to collect food (Fig. 2f). Settled individuals show a well-differentiated midgut gland, a pronounced trilobed foot, and a buccal cone with a mouth (Fig. 2f). Notably, adult scaphopods generally lack eyes and a distinct head.

**Fig. 3:**
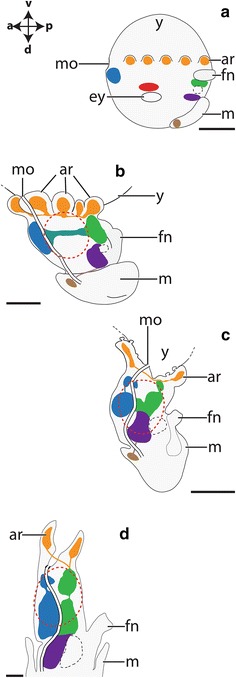
Ontogeny of the cephalopod *Idiosepius notoides.* All lateral views and dorsal (*d*)–ventral (*v*), anterior (*a*)–posterior (*p*) axes indicate the orientation. Core brain regions are highlighted, while innervation of peripheral target is omitted. The major ganglia are color coded: cerebral ganglia (*blue*), pedal ganglia (*green*), palliovisceral ganglia (*violet*), brachial ganglia (*orange*), and stellate ganglia (*brown*). The digestive tract is only indicated in the brain region, and the inner yolk duct and the entire external yolk sac are omitted for clarity. **a** Stage 19 embryos exhibit anlagen of all major ganglia. **b** The individual ganglia are connected in stage 23 embryos. Note that it is difficult to identify the origin of the brain mass connecting cerebral and pedal ganglia (herein labeled turquoise). **c** Stage 25 embryos exhibit a more centralized brain with the supraesophageal mass (*blue*), the posterior subesophageal mass (*violet*), the middle and anterior subesophageal mass (*green*), the brachial ganglia (*orange*), and the stellate ganglia (*brown*). **d** Hatchlings (stage 30) possess a CNS that largely resembles the one of adults. Please note that the stellate ganglia are not shown. *ar* arm, *ey* eye, *fn* funnel, *m* mantle, *mo* mouth and *y* yolk. *Scale bars* 150 µm

In the cephalopod *I. notoides*, cleavage only occurs on the cytoplasmic cap of the yolk-rich embryo (stages 2–13 according to [41]; reviewed in [42]). During the gastrulation process at stage 13, the outermost blastomere rows migrate below the inner blastomeres and a two-layered epithelium is formed on the yolk syncytium. Stage 18–19 individuals are roundish in shape and various organ systems are formed as placodes, among others the CNS, the arms, the funnel, the eyes, the mantle, and the arms (Fig. 3a). The brachial ganglia are located in the anlagen of the arms, the stellate ganglia are situated in the anterior portion of the mantle, and the optic ganglia are connected to both eyes (Fig. 3a). The cerebral ganglia develop dorsally to the mouth opening, the palliovisceral ganglia lie between the mantle and the statocysts, and the pedal ganglia are located ventrally to the statocysts. The dorso-ventral axis of stage 23 individuals is elongated compared to earlier stages (Fig. 3b). The esophagus is situated adjacent to the inner yolk duct and the individual ganglia connected to each other. Stage 25 embryos exhibit a more centralized brain and all individual central ganglia are termed brain masses herein in accordance with the classical literature (Fig. 3c). The cerebral ganglia give rise to the supraesophageal mass, the pedal ganglia develop into the anterior and middle subesophageal masses, and the palliovisceral ganglia are then termed posterior subesophageal mass [43]. Contrary, the peripheral stellate and brachial ganglia are still termed ganglia. The dorso-ventral body axis of stage 30 hatchlings is more elongated, and the CNS is more centralized than in earlier stages (Fig. 3d).

In this study, we describe hitherto unknown *Gsx* orthologs and their expression domains in the scaphopod *Antalis entalis* and the cephalopod squid *Idiosepius notoides* (Fig. 1). Our results question the widely assumed role of *Gsx* in patterning the foregut of the last common bilaterian ancestor and highlight similarities as well as differences among mollusks, lophotrochozoans, and bilaterians.

## Methods

### Collection and culture of animals

Adults of the scaphopod *Antalis entalis* were collected from approximately 30 m depth by the staff of the research vessel *Neomys* off the coast of Roscoff (France). Individuals were immediately transferred into dishes filled with seawater (see also [39]). Spawning occurred spontaneously or was induced by heat shocks, i.e., individuals were exposed to alternating water temperatures. Unfertilized eggs were rinsed several times and fertilized with sperm. Early- and mid-stage trochophore larvae were cultured in Millipore-filtered seawater (MFSW) with 50 mg streptomycin sulfate and 60 mg penicillin G per liter MFSW. Early cleavage stages, metamorphic competent larvae, and settled individuals were cultured in MFSW without antibiotics. Water was changed every other day. Metamorphosis occurred spontaneously or was induced by adding shell-gravel from the collection site.

Adults of the pygmy squid *Idiosepius notoides* were dip-netted in the sea grass beds of Moreton Bay, Queensland, Australia. Embryos were cultured and staged as described previously [43]. Development from freshly laid fertilized eggs (stage 1) to hatchlings (stage 30) takes 9–10 days at 25 °C.

### RNA extraction and fixation of animals

For *Antalis entalis*, a total of several hundred individuals of mixed developmental stages including early cleavage stages, trochophore larvae, metamorphic competent individuals, and early juveniles were collected and stored at −20 to −80 °C in RNAlater (Lifetechnologies, Vienna, Austria). RNA was extracted with a RNA extraction kit (Qiagen, Roermond, Netherlands) and stored at −80 °C.

For *Idiosepius notoides*, the egg jelly and chorion were removed from approximately 300 specimens covering freshly laid zygotes (stage 1) to hatchlings (stage 30). RNA was extracted using TriReagent according to the manufacturer’s instructions (Astral Scientific Pty. Ltd., Caringbah, Australia, see also [44]). Individuals of all the above-described developmental stages were fixed for in situ hybridization experiments as previously described [44].

### RNAseq and transcriptome assembly

Total RNA from pooled developmental stages of *Antalis entalis* was sequenced by Illumina technology (Eurofins, Ebersberg, Germany). Paired-end reads of an average read length of 100 bp were obtained and subsequently filtered (rRNA removal). Adapter and low-quality sequences were trimmed, normalized, and assembled *de novo* into contigs with the assembler Trinity [45].

RNA from developmental stages of *Idiosepius notoides* was sequenced by 454 and Illumina technology (both Eurofins) as described previously [44]. After filtering, the adapter and low-quality reads were trimmed, normalized, and assembled *de novo* by Eurofins (454 transcriptome) or using Trinity (Illumina transcriptome).

### Alignment and phylogenetic analysis

**Fig. 4:**
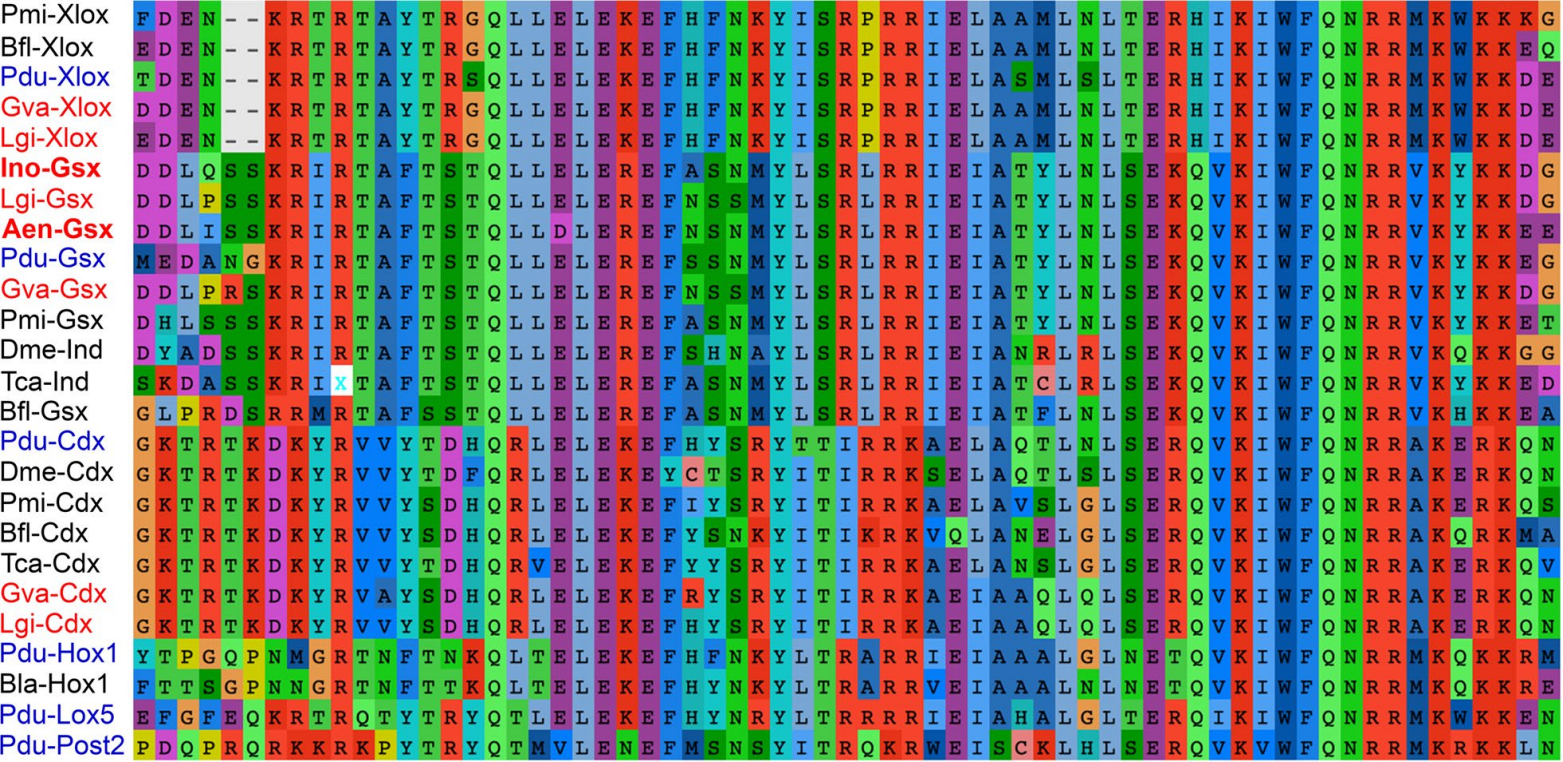
Alignment of amino acid sequences of bilaterian *ParaHox* and *Hox* gene orthologs. The Gsx amino acid sequences of *Idiosepius notoides* and *Antalis entalis* (*red-labeled*) cluster with their bilaterian orthologs. Molluscan Gsx orthologs are labeled in red and annelid orthologs in* blue*. GenBank or JGI accession numbers of amino acid sequences used for the alignment and the phylogenetic tree: Pmi-Xlox (*Patiria miniata*) AGK89734.1; Bfl-Xlox (*Branchiostoma floridae*) AAC39016.1; Pdu-Xlox (*P. dumerilii*) ACH87541.1; Gva-Xlox (*G. varia*) ADJ18240.1; Lgi-Xlox (*Lottia gigantea*) JGI e_gw1.80.260.1; Ino-Gsx (*I. notoides*) KT380894; Lgi-Gsx (*L. gigantea*) JGI e_gw1.30.54.1; Aen-Gsx (*A. entalis*) KT380895; Pdu-Gsx (*P. dumerilii*) ACH87538.1; Gva-Gsx (*G. varia*) KT380896; Pmi-Gsx (*P. miniata*) AGK89736.1; Dme-Ind (intermediate neuroblasts defective, *D. melanogaster*) AAC97116.1; Tca-Ind (intermediate neuroblasts defective, *Tribolium castaneum*) NP_001034494.1; Bfl-Gsx (*Branchiostoma floridae*) AAC39015.1; Pdu-Cdx (*P. dumerilii*) ABA29777.3; Dme-Cdx (*D. melanogaster*) AAF53923.1; Pmi-Cdx (*P. miniata*) AGK89735.1; Bfl-Cdx (*B. floridae*) AAC39017.1; Tca-Cdx (*T. castaneum*) NP_001034498.1; Gva-Cdx (*G. varia*) ADJ18241.1; Lgi-Cdx (*L.gigantea*) JGI estExt_Genewise1.C_sca_850005; Pdu-Hox1 (*P. dumerilii*) AFJ91921.1; Bla-Hox1 (*B. lanceolatum*) ACJ74382.1. Pdu-Lox5 (*P. dumerilii*) AFJ91925.1; Pdu-Post2 (*P. dumerilii*) AFJ91927.1

**Fig. 5:**
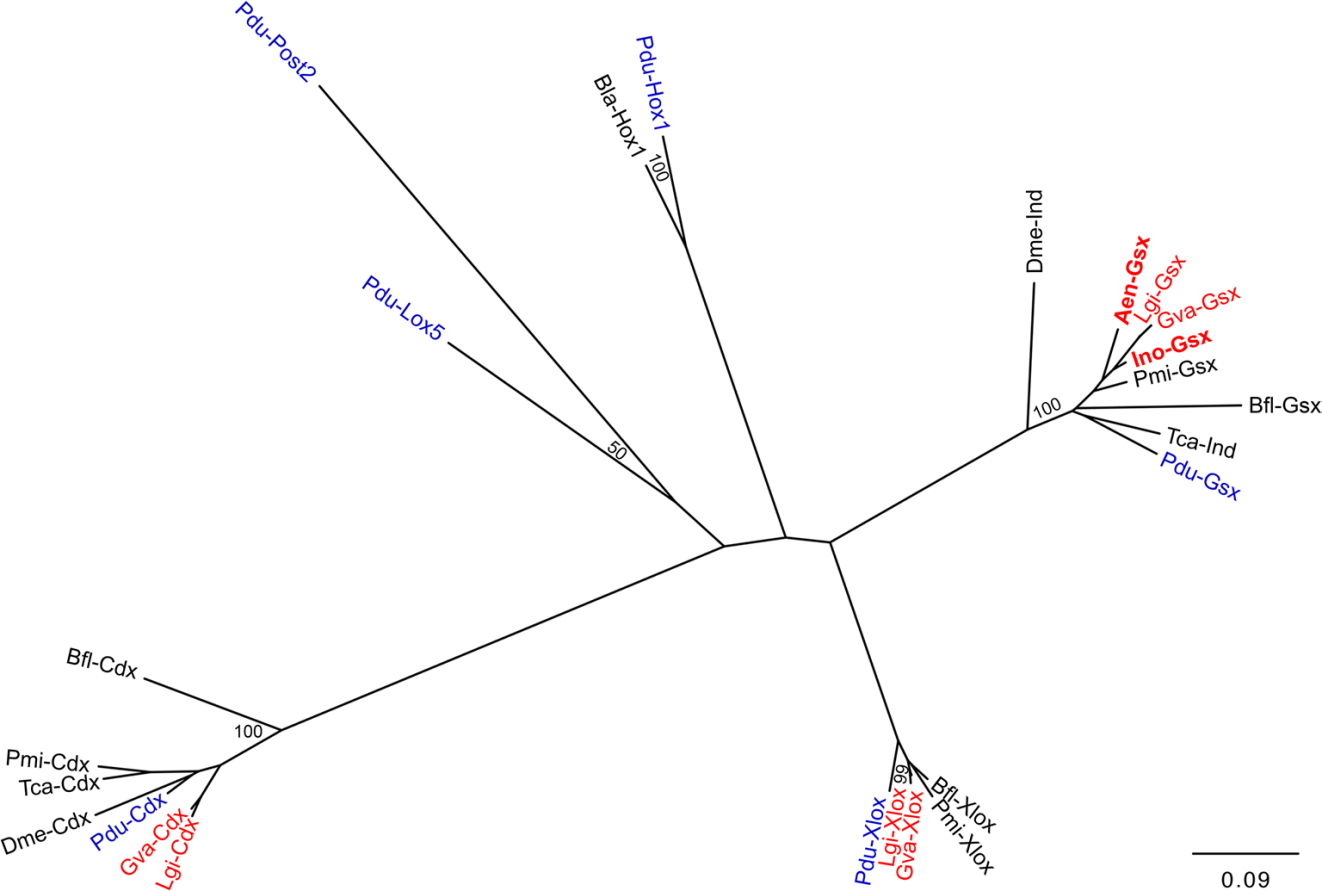
Phylogenetic analysis including the amino acid sequences of bilaterian *ParaHox* and *Hox* gene orthologs. This phylogenetic analysis of amino acid sequences of bilaterian orthologs of *Gsx*, *Cdx*, *Xlox*, and *Hox1* genes confirms the identity of *Aen*-*Gsx* and *Ino*-*Gsx* (*red-labeled*/*bold*). Molluscan Gsx orthologs are labeled in* red* and annelid orthologs in* blue*. Percentage of bootstrap support is shown when over 50 % and only for the major clades. See capture of Fig. 4 for accession numbers

Known amino acid sequences of bilaterian *Gsx* orthologs were retrieved from the National Center for Biotechnology Information (NCBI) and used in BLAST searches against both assembled transcriptomes. Amino acid sequences were aligned using ClustalX v.2.0 [46], trimmed by hand with the program AliView [47], and only conserved regions were retained (Fig. 4; untrimmed alignments are available upon request). This alignment was used to construct the neighbor-joining tree shown in Fig. 5 using the JTT matrix with 1000 bootstrap replicates within the Phylip v.3.695 [48] suite of programs.

### Molecular isolation of RNA transcripts

First-strand cDNA synthesis of the RNA pooled from different developmental stages of *Antalis entalis* and *Idiosepius notoides*, respectively, was carried out by reverse transcription using the First-strand cDNA Synthesis Kit for rt-PCR (Roche Diagnostics GmbH, Mannheim, Germany). Identified *Gsx* orthologs of *A. entalis* and *I. notoides* were used to design gene-specific primers, and PCR products were size-fractioned by gel electrophoresis. Gel bands of the expected length were excised and cleaned up using a QIAquick Gel Extraction Kit (QIAgen, Hilden, Germany). By insertion into pGEM-T Easy Vectors (Promega, Mannheim, Germany), cleaned-up products were cloned. Plasmid minipreps were grown overnight, cleaned up with the QIAprep Spin MiniprepKit (QIAgen), and sent for sequencing. The sequenced minipreps matched both transcripts identified as *Aen*-*Gsx* and *Ino*-*Gsx* in the phylogenetic analysis (Figs. 4, 5).

### Probe synthesis and whole-mount in situ hybridization

Riboprobe templates were amplified via standard PCR from miniprepped plasmids using M13 forward and reverse primers. *In vitro* transcription reactions were performed with these templates, digoxigenin-UTP (DIG RNA Labeling Kit, Roche Diagnostics) and SP6/T7 polymerase (Roche Diagnostics GmbH) for the syntheses of antisense riboprobes according to the manufacturer’s instructions. For whole-mount in situ hybridization experiments, specimens were rehydrated into PBT (PBS + 0.1 % Tween-20) and treated with Proteinase-K (25 µg/ml for *Idiosepius notoides* and 45 µg/ml for *Antalis entalis*) in PBT at 37 °C for 10 min. Specimens were prehybridized in hybridization buffer for 4 h at 50 °C (*A. entalis*) or 65 °C (*I. notoides*), and hybridization with a probe concentration of 0.5 μg/ml (*I. notoides*) to 1 μg/ml (*A. entalis*) was carried out overnight at 50 °C (*A. entalis*) or 65 °C (*I. notoides*). For *A. entalis* as well as *I. notoides*, a minimum of 20 individuals per stage were investigated, and negative controls were carried out with sense probes for all genes and developmental stages. The majority of whole-mount preparations were cleared in a solution of benzyl benzoate/benzyl alcohol (2:1), mounted on objective slides, and analyzed. Preparations were documented with an Olympus BX53 Microscope (Olympus, Hamburg, Germany). In addition, scaphopod developmental stages were scanned with a Leica confocal SP5 II microscope (Leica Microsystems, Wetzlar, Germany) using bright-field, autofluorescence, and reflection mode scans [49]. If necessary, images were processed with Adobe Photoshop 9.0.2 software (San Jose, CA, USA) to adjust contrast and brightness.

### Histology

After in situ hybridization experiments, developmental stages of *Antalis entalis* were post-fixed in 100 % EtOH and embedded in agar low viscosity resin (Agar Scientific, Essex, United Kingdom). Specimens were semithin sectioned with a diamond knife (Histo Jumbo Diatome) at a thickness of 0.5 µm with an ultramicrotome (Leica EM UC6, Wetzlar, Germany). Sections were mounted on objective slides, stained with Eosin using standard histological protocols, and covered with cover slips. Alternatively, after in situ hybridization, specimens were embedded in O.C.T. medium (VWR, Vienna, Austria) and cut into 15–30 µm cryosections with a cryotome (Leica CM 3050S). Sections were stained with Dapi (Sigma-Aldrich, St. Louis, MO, USA) and Cellmask Green plasma membrane stain (ThermoFisher, Waltham, MA, USA) in order to stain cell nuclei and cell membranes. Sections were mounted in Fluoromount G (Southern Biotech, Birmingham, Alabama, USA) and covered with cover slips. Semithin as well as cryotome sections was documented with an Olympus BX53 Microscope (Olympus).

### Statement of ethical approval

Developmental stages and adults of the pygmy squid *Idiosepius notoides* were collected, anesthetized, and fixed according to internationally recognized standards (University of Queensland Animal Welfare Permit No. 158/09 “The cultivation of *Idiosepius* (pygmy squid) for studies in developmental biology” to BMD).

## Results

### *Aen*-*Gsx* expression in developmental stages of the scaphopod *Antalis entalis*

The alignment of multiple amino acid sequences shows that *Aen*-*Gsx* and *Ino*-*Gsx* exhibit high sequence similarity with their bilaterian orthologs (Fig. 4). *Aen*-*Gsx* as well as *Ino*-*Gsx* clusters with their bilaterian orthologs in the phylogenetic analysis (Fig. 5).

**Fig. 6:**
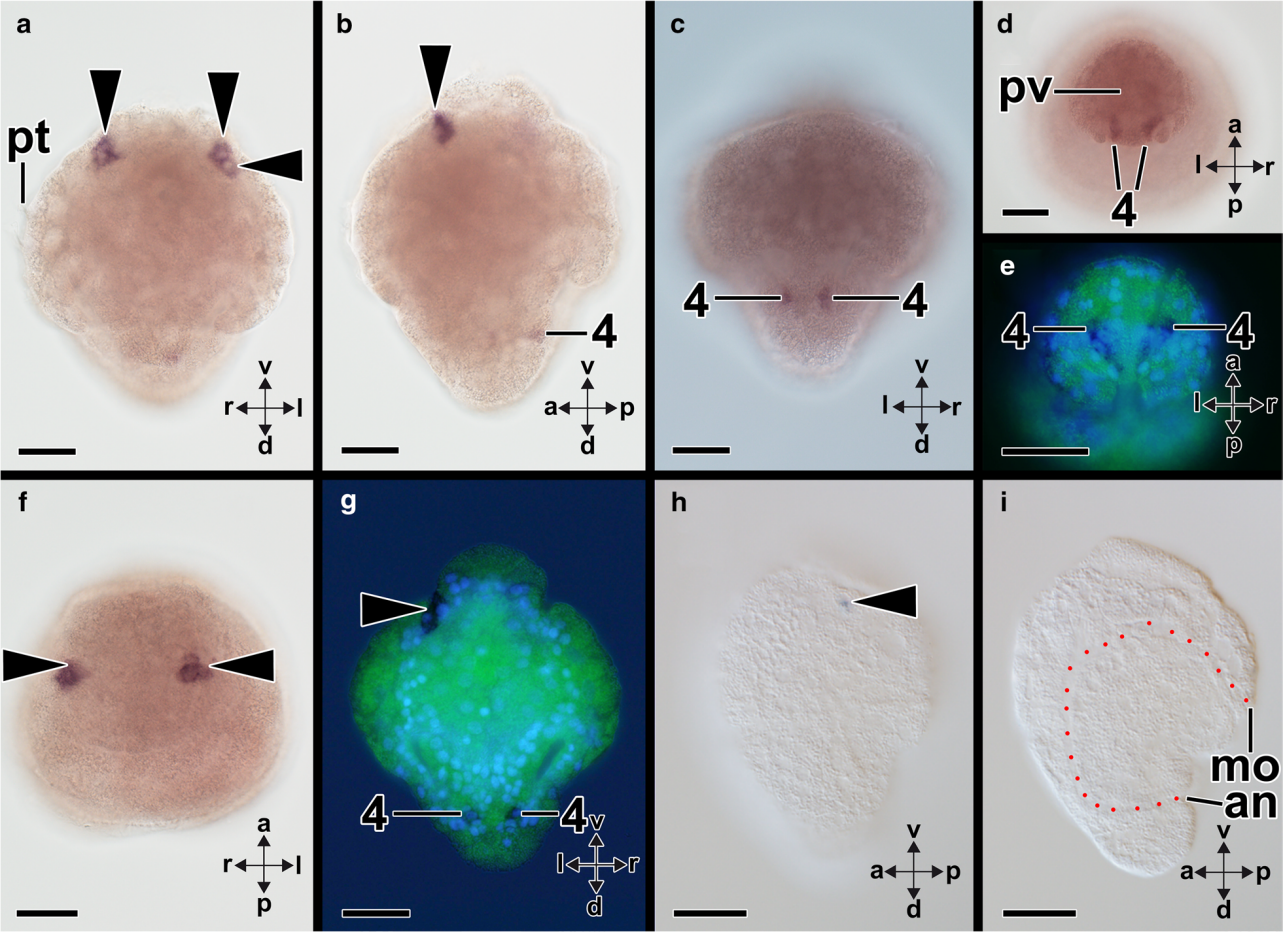
Expression of *Aen*-*Gsx* in early-stage trochophore larvae (14 hpf) of the scaphopod *Antalis entalis.* Dorsal (*d*)–ventral (*v*), anterior (*a*)–posterior (*p*), and left (*l*)–right (*r*) axes indicate the orientation. **a**–**d**, **f**, **h**, **i** are whole-mount preparations, while **e** and **g** are cryosections (cell membranes stained green, cellmask/cell nuclei labeled *blue*, Dapi). **a** Each two cells (*arrowheads*) express *Aen*-*Gsx* on both sides of the episphere. **b**–**e** Each one cell (4) expresses *Aen*-*Gsx* bilaterally to the anus. **f** Four *Aen*-*Gsx*-expressing cells (*arrowheads*) are located in episphere. **g** *Aen*-*Gsx*-expressing cells on the left side of the episphere (both cells on the right side are on another cryosection) and close to the anus (4). **h, i** BBA cleared specimen highlighting the position of mouth (*mo*), anus, and digestive tract (*red stippled line*) in relation to *Aen*-*Gsx*-expressing cells (*arrowhead*). *an* anus, *pt* prototroch. *Scale bars* 50 µm

**Fig. 7:**
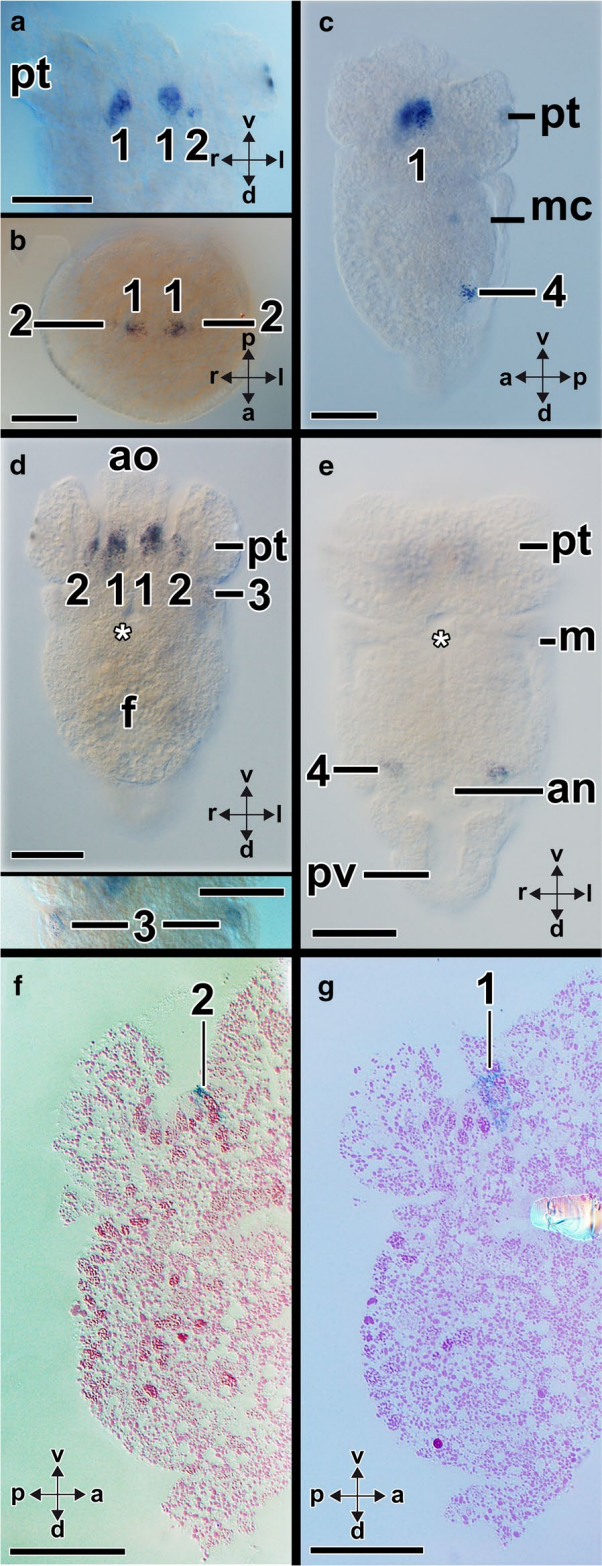
Expression of *Aen*-*Gsx* in mid-stage trochophore larvae of the scaphopod *Antalis entalis.* Dorsal (*d*)–ventral (*v*), anterior (*a*)–posterior (*p*), and left (*l*)–right (*r*) axes indicate the orientation. *Asterisks* mark the mouth opening. **a** Two cells (*1*) of the apical organ express *Aen*-*Gsx* in a 19 hpf old larva. Another two cells (*2*) faintly express *Gsx* and are located laterally to the former ones (the *right* cell is located in another cryotome section). **b** Apical view of 19 hpf old, same staged specimen showing both cells (*1*) in the apical organ and faintly both lateral cells 2. **c** Lateral view of a 21 hpf mid-stage trochophore larva. In this optical section, both *Aen*-*Gsx*-expressing cells in the apical organ and both *Aen*-*Gsx*-expressing cells (*4*) on both sides below the mantle lateral to the anus. **d** Same specimen as shown in **c** showing a pair of *Aen*-*Gsx*-expressing cells (*3*) on the latero-posterior-most side of the mantle. Both cells in the apical organ (*1*) as well as lateral to the latter (*2*) express *Aen*-*Gsx*. *Inset*: magnification of the *Aen*-*Gsx*-expressing cells (*3*) in a same staged specimen. **e** Same specimen as shown in **c**, **d** with both *Gsx*-expressing cells (*4*) on both sides below the mantle lateral to the anus. **f** This histological section shows a lateral *Aen*-*Gsx*-expressing cell (*2*) that penetrates the epidermis. **g** This histological section shows an *Aen*-*Gsx*-expressing cell (*1*) belonging to the apical organ. *an* anus, *f* foot, *m* mantle, *mc* mantle cavity and *pt* prototroch. *Scale bars* 50 µm

**Fig. 8:**
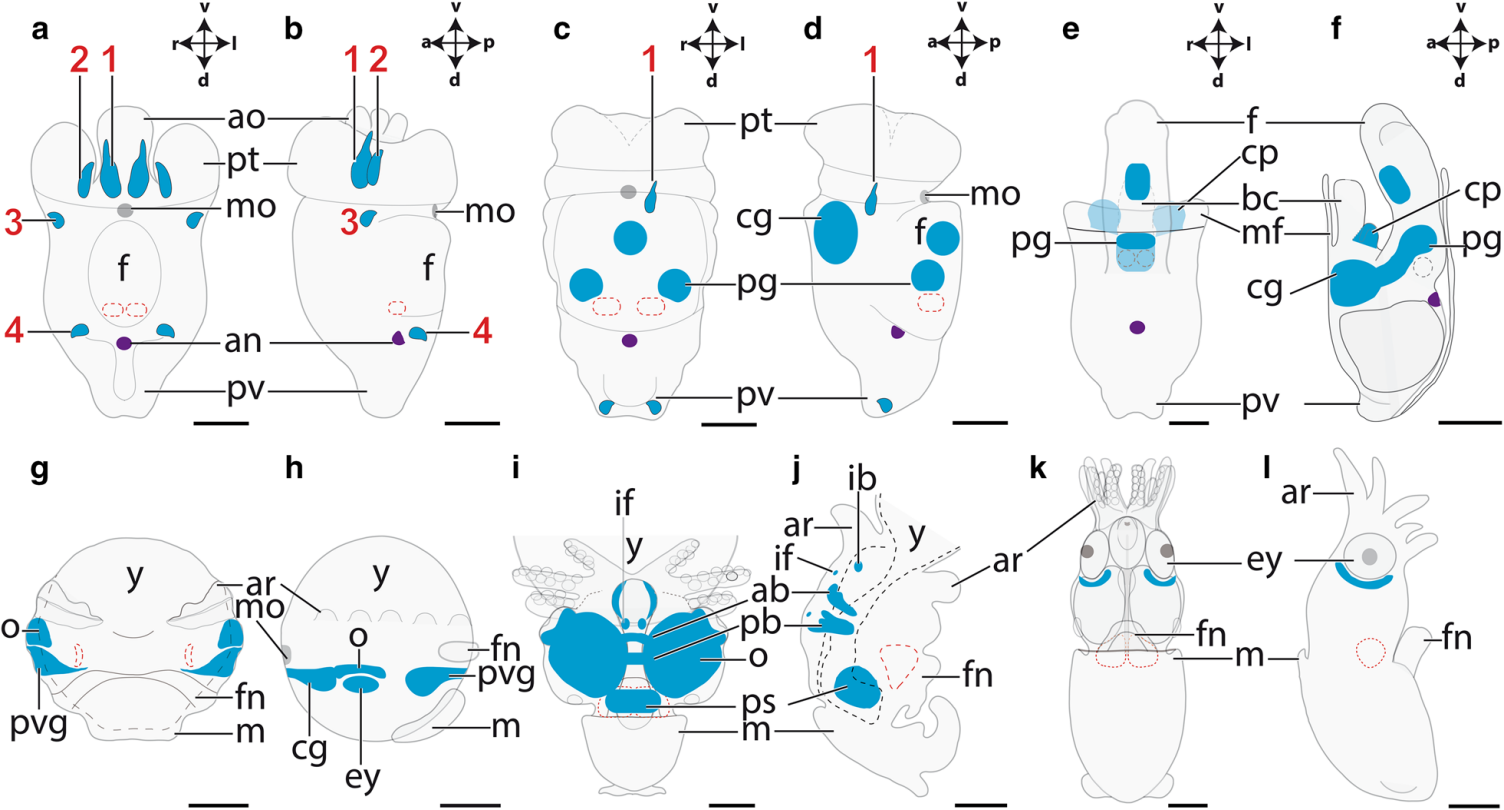
Summary of *Gsx* expression (*blue*) during the development of the scaphopod *Antalis entalis* and the cephalopod *Idiosepius notoides.* Dorsal (*d*)–ventral (*v*), anterior (*a*)–posterior (*p*), and left (*l*)–right (*r*) axes indicate the orientation and are the same in each of the six columns. Shown *Gsx*-expressing cell somata do not represent absolute numbers. **a–f** Sketch drawing depicting a mid-stage trochophore (**a**, **b**), a late-stage trochophore (**c**, **d**), and a postmetamorphic (settled) individual (**e**, **f**) of *Antalis entalis*. *Gsx*-expressing cell somata are labeled with *red numbers* 1–4 (c.f. Figures 6, 7). **g–l** Sketch drawing depicting developmental of stage 19 (**g**, **h**), stage 25 (**i**, **j**), and stage 28 (**k**, **l**, yolk sac removed) of the pygmy squid *Idiosepius notoides*. *ab* anterior basal lobe, *ao* apical organ, *ar* arm, *bc* buccal cone, *cg* cerebral ganglion, *cp* captacula, *ey* eye, *f* foot, *fn* funnel, *ib* inferior buccal lobe, *if* inferior frontal lobe, *m* mantle, *mf* mantle fold, *mo* mouth, *o* optic ganglion/lobe, *pb* posterior basal lobe, *pg* pedal ganglion, *pt* prototroch, *pv* pavilion, *pvg* palliovisceral ganglion, *ps* posterior subesophageal mass and *y* yolk. *Scale bars* **a**–**f** 50 µm, **g**–**l** 150 µm

**Fig. 9:**
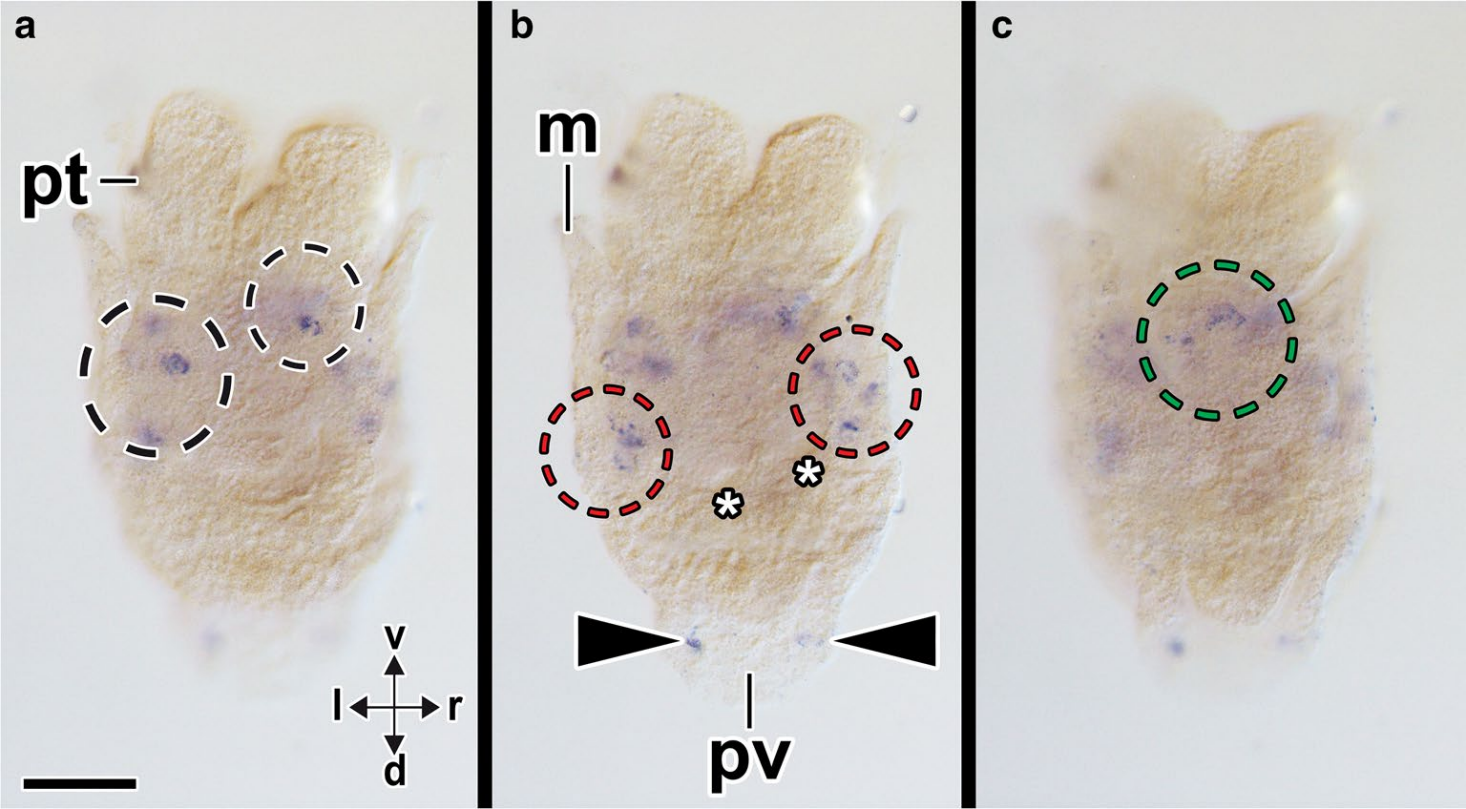
Expression of *Aen*-*Gsx* during late-stage trochophores of the scaphopod *Antalis entalis*. Dorsal (*d*)–ventral (*v*) and left (*l*)–right (*r*) axes indicate the orientation. The statocysts are labeled with asterisks. Optical sections from anterior (**a**) to posterior (**b**) in a 63 hpf old trochophore (all with same orientation and *scale bar* as indicated in **a**). **a** One cell cluster on each side expresses *Aen*-*Gsx* in the region of the cerebral ganglia (*black dashed circles*). **b** Two cell clusters (*red dashed circles*) express *Aen*-*Gsx* ventro-laterally to the statocysts, in the region of the prospective pedal ganglia. A pair of *Aen*-*Gsx*-expressing cells is located in the region of the pavilion (pv). **c** *Aen*-*Gsx* is expressed in the ventral portion of the foot (*green dashed circle*). *m* mantle, *pt* prototroch. *Scale bar* 50 µm

**Fig. 10:**
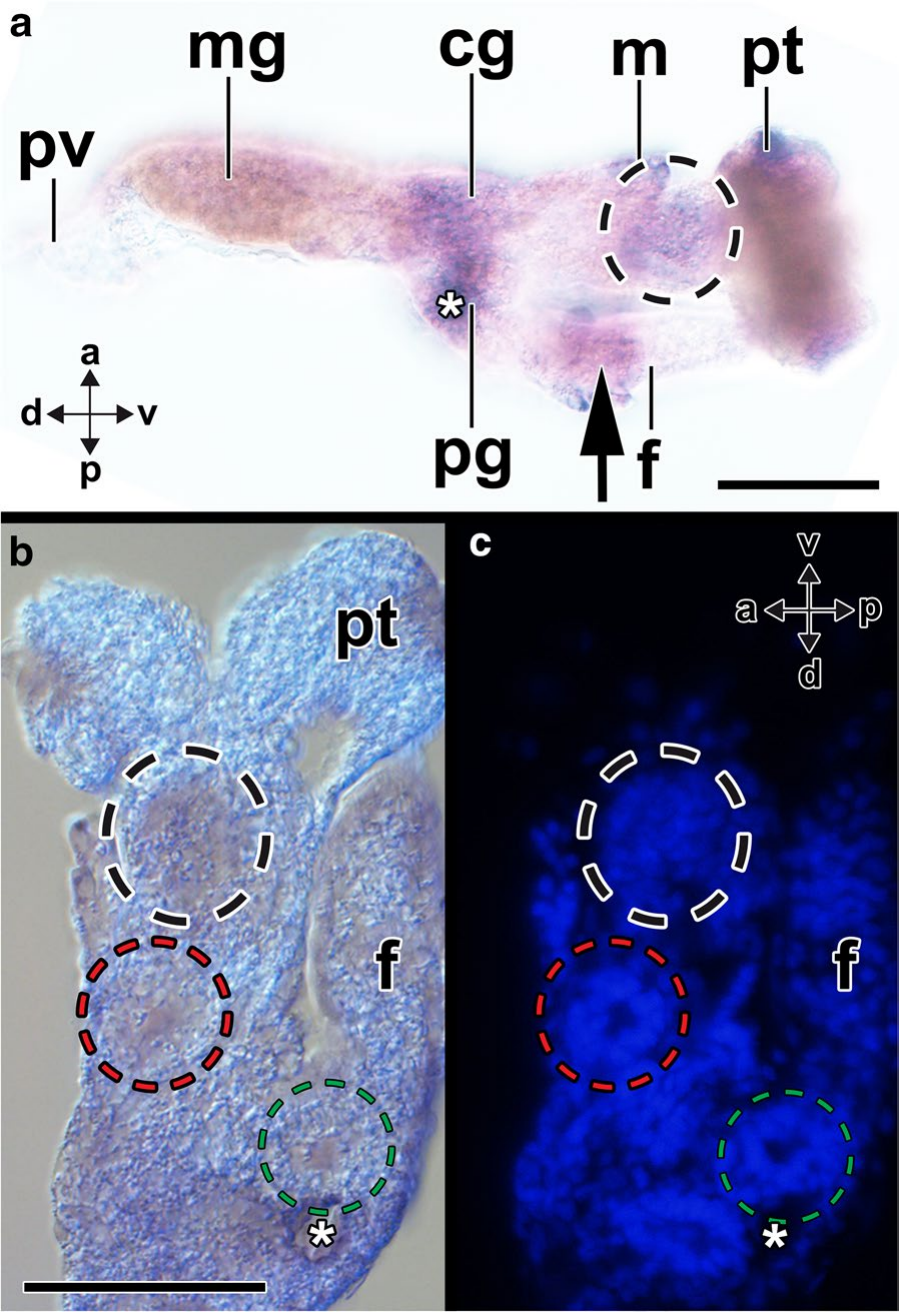
Expression of *Aen*-*Gsx* in metamorphic competent larvae (70 hpf) of the scaphopod *Antalis entalis*. In all three cryosections, anterior (*a*)–posterior (*p*), dorsal (*d*)–ventral (*v*) and left (*l*)–right (*r*) axes indicate the orientation. The statocysts are labeled with asterisks. **a** *Aen*-*Gsx* is expressed in the region of the cerebral (*cg*) and pedal (*pg*) ganglia as well as in the region of the anterior foot (*f*) (*arrow*). Note the *Aen*-*Gsx* expression in the region of the nascent captacula (black dashed circle). **b**, **c** *Aen*-*Gsx* expression in the region of the nascent captacula (*black dashed circle*), the cerebral ganglia (*red dashed circle*), and pedal ganglia (*green dashed circle*). The bright-field micrograph (**b**) and the nuclear counterstain (*blue*, Dapi) show the same individual of the same orientation and size. Note the perikaryal layers around the cerebral and pedal ganglia shown in **c**. *m* mantle, *mg* midgut gland, *pt* prototroch, *pv* pavilion. *Scale bars* 100 µm

**Fig. 11:**
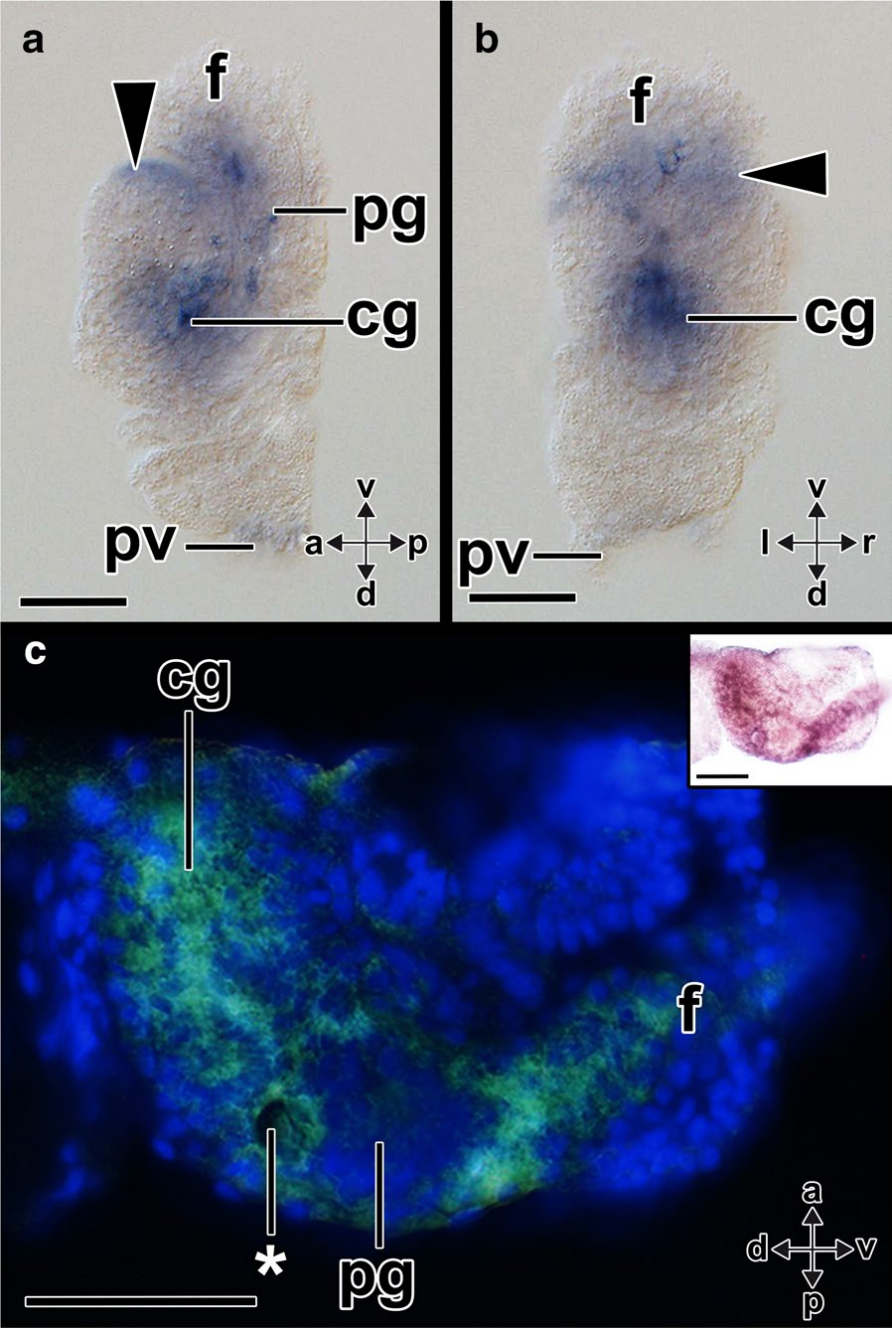
Expression of *Aen*-*Gsx* during postmetamorphic development (114 hpf) of the scaphopod *Antalis entalis*. Anterior (*a*)–posterior (*p*), dorsal (*d*)–ventral (*v*), and left (*l*)–right (*r*) axes indicate the orientation. The statocysts are labeled with asterisks. **a**, **b** *Aen*-*Gsx* expression in the region of the cerebral (*cg*) and pedal ganglia (*pg*) as well as the ventral foot (*f*) and the ventral region of the nascent captacula (*arrowheads*). **c** Inverted bright-field image of *Aen*-*Gsx* expression (*green*) with nuclear counterstain (*blue*, Dapi) of a cryosection (see *inset* for original stain). *Aen*-*Gsx* is expressed in the cerebral and pedal ganglia as well as the foot. Faint staining is also visible in the region of the nascent captacula. *pv* pavilion. *Scale bars* 50 µm

*Aen*-*Gsx* is first expressed in two cells in the episphere of early-stage trochophore larvae (14 hpf) (arrowheads in Fig. 6a, b, f–h). In addition, each one *Aen*-*Gsx*-expressing cell is located laterally to the anus (“4” in Fig. 6c–e, g). In mid-stage trochophore larvae (19 hpf), *Aen*-*Gsx* is expressed in two flask-shaped cells of the apical organ (“1” in Figs. 7a, b, 8a, b) and two lateral cells (“2” in Figs. 7a, b, 8a, b). Mid-stage trochophore larvae at 21 hpf also exhibit both above-mentioned groups of cells (Fig. 7c, d). While two *Aen*-*Gsx*-expressing cells are located in the apical organ (“1” in Figs. 7d, g, 8a, b), both lateral *Aen*-*Gsx*-expressing cells do not appear to belong to the latter (“2” in Figs. 7d, f, 8a, b). Another pair of *Aen*-*Gsx*-expressing cells is present on the posterolateral side of the mantle (“3” in Figs. 7d, 8a, b) and below the mantle laterally to the anus (“4” in Figs. 7e, 8a, b). In late-stage trochophore larvae, two clusters of *Aen*-*Gsx*-expressing cells are present at the base of both captacula, in a region where the future cerebral ganglia develop (Fig. 8c, d; black dashed circle in Fig. 9a). Two additional clusters of *Aen*-*Gsx*-expressing cells are located ventro-laterally to the statocysts (Fig. 8c, d; red dashed circle in Fig. 9b). Two flask-shaped *Aen*-*Gsx*-expressing cells are located in the region of the pavilion (Fig. 8c, d; arrowheads in Fig. 9b). Another group of *Aen*-*Gsx*-expressing cells is located in the ventral portion of the foot (Fig. 8c; green dashed circle in Fig. 9c). In some individuals, one or two flask-shaped *Aen*-*Gsx*-expressing cells are visible in the region close to the cerebral ganglia (data not shown). In metamorphic competent trochophore larvae, each one *Aen*-*Gsx*-expressing cell cluster is situated ventro-laterally to the statocysts in the region of the pedal ganglia (green dashed circles in Fig. 10b, c) and in the region of the cerebral ganglia (red dashed circles in Fig. 10b, c). Other *Aen*-*Gsx*-expressing cell clusters are located in the region of the nascent captacula (black dashed circles in Fig. 10). Postmetamorphic specimens exhibit a similar distribution of *Aen*-*Gsx*-expressing cells in the regions of the cerebral and pedal ganglia (Figs. 8e, f, 11a–c). *Aen*-*Gsx*-expressing cells are also present in the region of the nascent captacula (arrowheads in Fig. 11a, b) and in the ventral foot region (Figs. 8e, f, 11a–c).

### *Ino*-*Gsx* expression in *Idiosepius notoides*

**Fig. 12:**
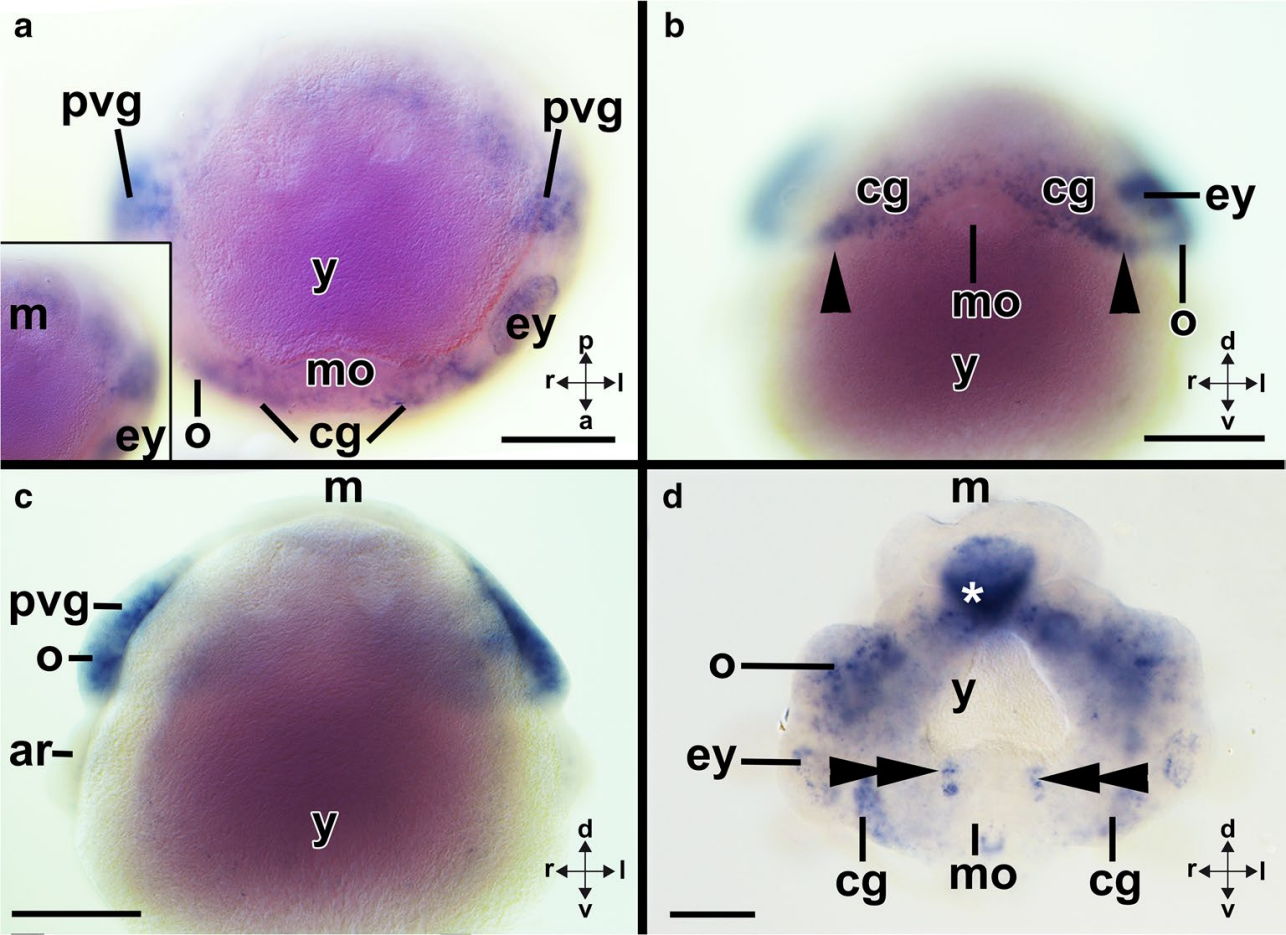
Expression of *Ino*-*Gsx* during development of the cephalopod *Idiosepius notoides.* Dorsal (*d*)–ventral (*v*), anterior (*a*)–posterior (*p*), and left (*l*)–right (*r*) axes indicate the orientation. **a** *Gsx* is expressed in the region of the cerebral (*cg*) and palliovisceral ganglia (*pvg*) of stage 19 individuals. Inserted image depicts other optical section highlighting the mantle (*m*). **b** In addition to the optic ganglia, *Ino*-*Gsx* is expressed in the cerebral ganglia that expand anteroventrally in direction of the eyes of stage 19 individuals (*arrowheads*). **c** Optical section through the middle of the specimen shown in **a** and **b** which depicts the connection of the optic and palliovisceral ganglia. **d** Stage 22 individuals express *Ino*-*Gsx* in two domains of the cerebral ganglia, among others in the region of the future buccal lobes (*double arrowheads*). Note the unspecific signal in the intestines of this specimen (*asterisk*). Note unspecific staining (*purple*) in yolk sac (removed in **d**). *ar* arm, *ey* eye, *mo* mouth, *o* optic ganglion/lobe, *y* yolk. *Scale bars* 150 µm

**Fig. 13:**
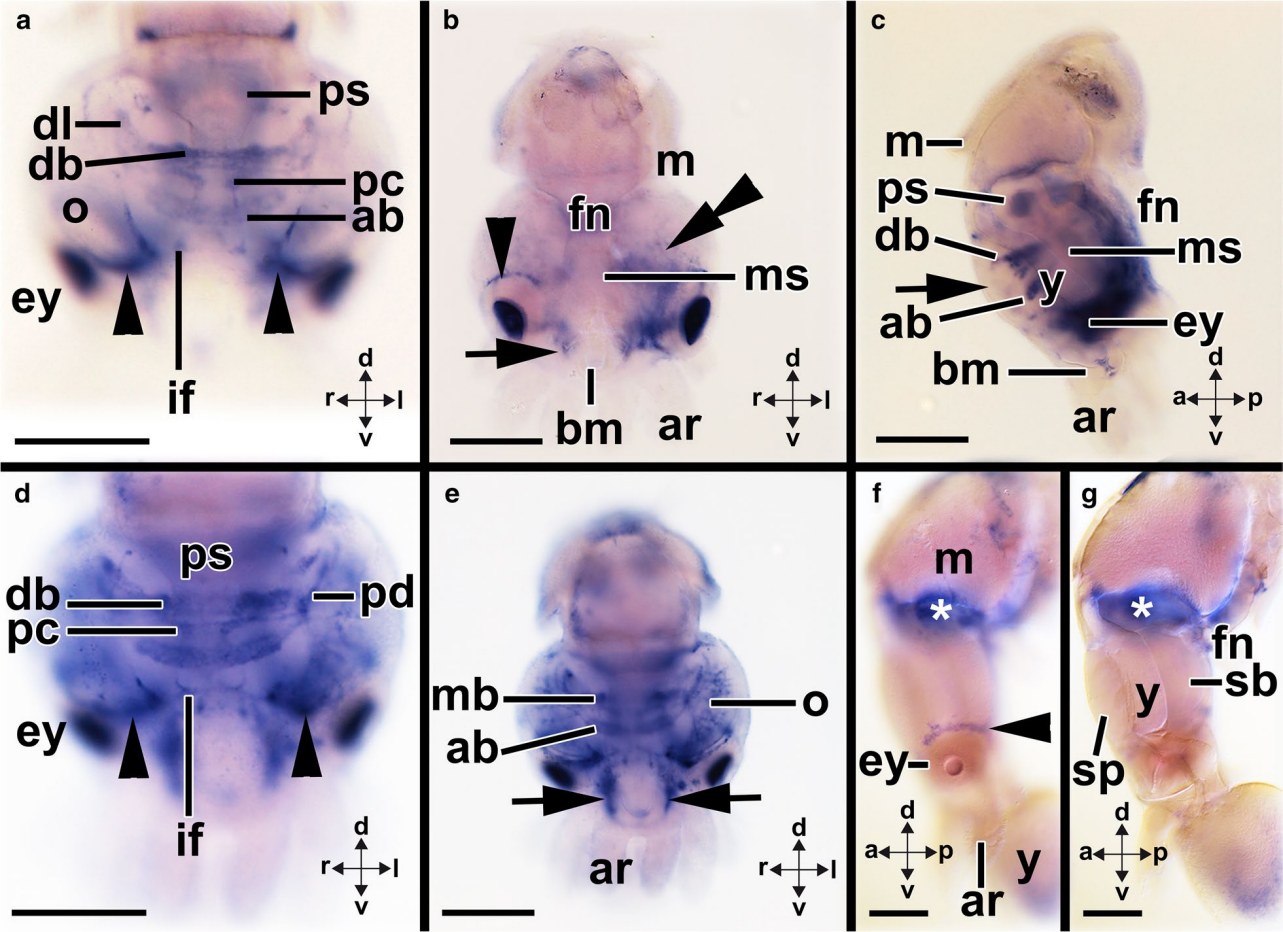
Expression of *Ino*-*Gsx* during late development of the cephalopod *Idiosepius notoides.* Dorsal (*d*)–ventral (*v*), anterior (*a*)–posterior (*p*), and left (*l*)–right (*r*) axes indicate the orientation. Yolk sac removed in **a**–**e**. **a** Cephalic region of a stage 25 individual which expresses *Ino*-*Gsx* widely in the supraesophageal mass including the inferior frontal (*if*), anterior basal (*ab*), dorsal basal (*db*), dorsolateral (*dl*), and precommissural lobes (*pc*). *Ino*-*Gsx* expression is also observed around the eyes (*arrowheads*). **b** Same specimen as seen in **a** with *Ino*-*Gsx* expression around the eye (*arrowhead*), with faint expression on the lateral sides of the buccal region (*arrow*) and in the optic lobes (*double-arrowhead*). Note the lack of *Ino*-*Gsx* expression in the middle subesophageal mass (*ms*). **c** Same specimen as seen in **a** and **b** with *Ino*-*Gsx* expression in the posterior subesophageal mass (*ps*) but not in the middle subesophageal mass. Note the lack of *Ino*-*Gsx* expression in the vertical lobe (*arrow*) that is the anterior-most brain region. **d** *Ino*-*Gsx* is strongly expressed in the supraesophageal mass of stage 26 individuals including the peduncle lobes (*pd*) (here only cephalic region visible). Note the *Ino*-*Gsx* expression domain around the eyes (*arrowheads*). **e** Same specimen as seen in H with strong expression in the optic lobes and, among others, in the median basal lobes (*mb*). Note the strong *Ino*-*Gsx* expression on the lateral sides of the buccal mass (*arrows*). **f** Stage 28 individuals up to hatchlings express *Ino*-*Gsx* in the circular domain around the eyes (*arrowhead*); however, expression is lacking in the CNS. Note the unspecific staining in the statocysts. **g** Optical section along the sagittal plane of the same specimen as shown in **j**. No *Ino*-*Gsx* expression is visible in the supraesophageal (*sp*) or subesophageal masses (*sb*) of the CNS. Note the unspecific staining in the statocysts. *ar* arm, *bm* buccal mass, *ey* eye, *fn* funnel, *mo* mouth, *o* optic lobe, *y* yolk. *Scale bars* **a**–**e** 150 µm and **f**, **g** 200 µm

In stage 19–20 individuals, *Ino*-*Gsx* is expressed in the region of the optic and palliovisceral ganglia (Figs. 8g, h, 12a–c). The cerebral ganglia, which are located dorsally to the mouth and expand anteroventrally in direction of the eyes, also express *Ino*-*Gsx* (Fig. 8h; arrowheads in Fig. 12b). In subsequent developmental stages, the expression domains remain the same and stage 23 individuals express *Ino*-*Gsx* in the optic and palliovisceral ganglia (Fig. 12d). The expression domain in the cerebral ganglia is relatively smaller compared to the domain reported for previous stages, and it is restricted to two patches ventro-laterally to the eye and close to the forming buccal mass (double arrowheads in Fig. 12d). In subsequent developmental stages, individual lobes of the supraesophageal mass as well as the posterior subesophageal mass and the optic lobes express *Ino*-*Gsx* (Fig. 13a–e). In the supraesophageal mass of stage 25 individuals, *Ino*-*Gsx* expression occurs in the inferior frontal and precommissural lobes as well as in the anterior basal and posterior basal lobes including the dorsal basal and dorsolateral lobes (Figs. 8i, j, 13a–c). In addition, *Ino*-*Gsx* is still expressed around the eyes and laterally of the buccal mass. This area might correspond to the region where the inferior buccal lobes develop (Figs. 8i, j, 13a, b). No expression was observed in the vertical, subvertical, and the superior frontal lobes or the anterior or middle subesophageal masses (Figs. 8i, j, 13a–c). Compared to stage 25 individuals, stage 26 individuals strongly express *Ino*-*Gsx* in their optic lobes (Figs. 8i, j, 13a–c). In addition, lobes of the supraesophageal mass such as the peduncle lobes or the buccal lobes express *Ino*-*Gsx* (Figs. 8i, j, 13a–c). Stronger *Ino*-*Gsx* expression is also observed laterally of the buccal mass, most likely corresponding to the inferior buccal lobes (Fig. 13e). Subsequent developmental stages until hatching only express *Ino*-*Gsx* around the eyes but not in the CNS (Figs. 8k, l, 13f, g).

## Discussion

### *Gsx* does not pattern the digestive tract of scaphopods and cephalopods

To date, it is commonly hypothesized that the digestive tract of the last common bilaterian ancestor expressed *Gsx* in a collinear fashion together with the two other *ParaHox* genes, *Cdx* and *Xlox* [1, 6, 12, 20, 50]. This hypothesis is seemingly corroborated by the fact that among the Lophotrochozoa, the annelids *Platynereis dumerilii* and *Nereis virens*, as well as the gastropod *Gibbula varia*, express *Gsx* in their anterior digestive tract (Table 1; [12–14]). Our results for the scaphopod *Antalis entalis* and the cephalopod *Idiosepius notoides*, however, show that this is not the case for all mollusks, and therefore, neither for all lophotrochozoans, a scenario that was already suggested by data on the annelid *Capitella teleta* ([15]; Table 1). Moreover, all ecdysozoan representatives investigated lack *Gsx* expression in their digestive tract, and among the deuterostomes investigated, only the hemichordate *Ptychodera flava* expresses *Gsx* around the blastopore [7]. The lack of *Gsx* expression in the foregut of the other deuterostomes has been explained by the fate of the blastopore that does not transform into the definite mouth in deuterostomes as it does in protostomes, but, instead, into the anus [6]. Accordingly, the latter hypothesis would argue for *Gsx* expression in the deuterostome hindgut which, however, appears to be absent ([50]; Table 1). It is important to mention that *Gsx* orthologs have either not been found or are indeed absent in representatives of the Acoelomorpha, which are characterized by having a single mouth/anus opening in their digestive tract and may form the sister taxon to all remaining Bilateria (the so-called Nephrozoa; [51, 52]; but see [53] for a controversial view). In cnidarians, *Gsx* is endodermally expressed in the planula larva of *Nematostella vectensis*, *Clytia hemisphaerica*, and *Podocoryne carnea* [9–11]. In the coral *Acropora millepora, Gsx* is expressed in the ectoderm of the planula larva [8]. Comparisons of the cnidarian and nephrozoan expression domains are difficult since mouth and digestive system cannot be easily homologized. Hence, the data currently available argue for a last common nephrozoan and probably also bilaterian ancestor without *Gsx* expression in the digestive tract and for a recruitment of *Gsx* into foregut patterning in selected lineages. Accordingly, the gastropod *G. varia* and the polychaete annelids *N. virens* and *P. dumerilii* have acquired *Gsx* expression in the foregut secondarily during evolution (Table 1). In contrast, other genes such as *Brachyury*, *Nkx2.1*, or *FoxA* appear to be evolutionary highly conserved in the digestive system within the Lophotrochozoa [54–59].

### *Gsx* is expressed in the anterior-most portion of the molluscan CNS

In contrast to the digestive tract, *Gsx* is consistently expressed in the anterior CNS of bilaterians and hence an ancestral role in CNS development was proposed (Table 1; [2]). Shared *Gsx* expression domains among mollusks are the cerebral ganglia that subsequently develop into the supraesophageal mass in cephalopods (present study; [12]). In scaphopod and gastropod larvae, the apical organ is located in the anterior-most region. In the scaphopod *Antalis entalis, Gsx* is expressed in two flask-shaped cells of this organ and in two cells that are located laterally to it but do not constitute a part of the apical organ (Fig. 4d). With two apical tuft cells and further putative sensory cells, the larva of the gastropod *Gibbula varia* possesses more *Gsx*-expressing cells in the apical organ than the one of *A. entalis* (present study; [12]). The flask-shaped *Gsx*-expressing cells of *A. entalis* do not appear to be homologous to any of the *Gsx*-expressing cell types of *G. varia* judging by their morphology. However, detailed ultrastructural studies and molecular fingerprints on the various cell types occurring in lophotrochozoan apical organs are necessary to further assess homologies in this organ on the cellular level. Among all metazoans with an apical organ (Cnidaria, Ambulacraria, and Lophotrochozoa), only both above-mentioned mollusks and the annelid *Platynereis dumerilii* possess *Gsx*-expressing cells in the apical organ, suggesting that *Gsx* has been recruited into the patterning of this sensory organ in lophotrochozoans only (Table 1; present study; [12, 13]).

*Gsx* expression has also been reported for the polychaete annelids *Nereis virens* and *Capitella teleta* [14, 15]. As far as known, both species lack an apical organ as do cephalopods as direct developers (present study; [3, 15]). The vertical lobe as the anterior-most portion of the cephalopod CNS does not express *Gsx* (Figs. 9d, 10). This resembles the expression patterns of other homeobox genes such as *Otx* or the *POU* genes which are consistently expressed in the gastropod cerebral ganglia and large parts of the cephalopod cerebral ganglia/supraesophageal mass but not in the vertical lobe [44, 60, 61]. The vertical lobe is considered an evolutionary innovation of coleoid cephalopods, i.e., all cephalopods except the nautiluses as basal cephalopod offshoots [62]. As an evolutionary younger brain region confined to coleoid cephalopods, the vertical lobe also differentiates relatively late during ontogeny compared to other brain regions [63]. Hence, the vertical lobe probably evolved after *Otx* expression domains had already been established in the supraesophageal mass of coleoid cephalopods.

### *Gsx* is expressed in the posterior portion of the molluscan CNS

*Idiosepius notoides* and *Antalis entalis* express *Gsx* in posterior portions of their CNS such as the scaphopod pedal ganglia and the cephalopod palliovisceral ganglia (the latter develop into the future posterior subesophageal mass). This is in contrast to the gastropod *Gibbula varia* and the annelid *Capitella teleta*, where *Gsx* expression is restricted to the anterior CNS [12, 15]. The scaphopod and cephalopod condition is, however, similar to the condition found in *Platynereis dumerilii* and certain vertebrates insofar that both mollusks and the polychaete express *Gsx* in more posterior regions of their nervous system. These domains comprise the scaphopod pedal ganglia, the cephalopod palliovisceral lobe/posterior subesophageal mass, the polychaete nerve cord, and the hindbrain of vertebrates [13, 24–27]. Interestingly, *Gsx* is also expressed in portions of the developing visual system of few representatives of all three bilaterian superphyla. The mollusks *I. notoides* and *Nereis virens*, the arthropods *Drosophila melanogaster*, as well as the teleost fish *Oryzias latipes,* express *Gsx* in portions of their visual system (Table 1; present study; [14, 17, 28]). Further studies on other bilaterian representatives are needed to assess if *Gsx* expression in the eyes and related brain regions may be an ancestral trait among nephrozoans or bilaterians.

## Conclusions

This study suggests that *Gsx* expression in the foregut is not a molluscan plesiomorphy and together with already published data argues against *Gsx* expression in the foregut of the last common bilaterian ancestor. It is therefore most likely that *Gsx* has been independently recruited into the development of the foregut in some lophotrochozoan representatives. *Gsx* is consistently expressed in the developing anterior nervous system of bilaterians, which is probably an apomorphy of Bilateria. In contrast to other metazoan taxa, *Gsx* expression was only found in the larval apical organ in lophotrochozoans, indicating that *Gsx* expression in the apical organ may be a lophotrochozoan synapomorphy.

## Abbreviations

a: Anterior
ab: Anterior basal lobe
*Aen*: *Antalis entalis*
*A. entalis*: *Antalis entalis*
an: Anus
ar: Arm
at: Apical tuft
ao: Apical organ
BBA: Benzyl benzoate
bc: Buccal cone
Bfl: *Branchiostoma floridae*
Bla: *Branchiostoma lanceolatum*
bm: Buccal mass
bp: Blastopore
brc: Brachial crown
c.f.: Confer
cg: Cerebral ganglia
cp: Captacula
CNS: Central nervous system
d: Dorsal
db: Dorsal basal lobe
dl: Dorsolateral lobe
Dme: *Drosophila melanogaster*
ep: Episphere
ey: Eye
f: Foot
fn: Funnel
*Gsx*: Genomic screened homeobox protein
*G. varia*: *Gibbula varia*
Gva: *Gibbula varia*
hpf: Hours after fertilization
hp: Hyposphere
ib: Inferior buccal lobe
i.e.: Id est
if: Inferior frontal lobe
ind: Intermediate neuroblasts defective
*Ino*: *Idiosepius notoides*
*I. notoides*: *Idiosepius notoides*
l: Left
Lgi: *Lottia gigantea*
m: Mantle
ma: Mantle apex
mb: Median basal lobe
mc: Mantle cavity
mf: Mantle fold
mg: Midgut gland
MFSW: Millipore-filtered seawater
mo: Mouth
ms: Middle subesophageal mass
NCBI: National Center for Biotechnology Information
o: Optic ganglion/lobe
p: Posterior
*P. dumerilii*: *Platynereis dumerilii*
Pdu: *Platynereis dumerilii*
pb: Posterior basal lobe
pc: Precommissural lobe
pd: Peduncle lobe
pg: Pedal ganglion
Pmi: *Patiria miniata*
ps: Posterior subesophageal mass
pt: Prototroch
pv: Pavilion
pvg: Palliovisceral ganglia
r: Right
sb: Subesophageal mass
sh: Shell
shf: Shell field
sp: Supraesophageal mass
Tca: *Tribolium castaneum*
v: Ventral
y: Yolk
